# Local Treatment of Colorectal Liver Metastases in the Presence of Extrahepatic Disease: Survival Outcomes from the Amsterdam Colorectal Liver Met Registry (AmCORE)

**DOI:** 10.3390/cancers16061098

**Published:** 2024-03-08

**Authors:** Hannah H. Schulz, Madelon Dijkstra, Susan van der Lei, Danielle J. W. Vos, Florentine E. F. Timmer, Robbert S. Puijk, Hester J. Scheffer, M. Petrousjka van den Tol, Birgit I. Lissenberg-Witte, Tineke E. Buffart, Kathelijn S. Versteeg, Rutger-Jan Swijnenburg, Martijn R. Meijerink

**Affiliations:** 1 Department of Radiology and Nuclear Medicine, Amsterdam University Medical Centers, 1081 HV Amsterdam, The Netherlands; 2 Department of Radiology and Nuclear Medicine, Noordwest Ziekenhuisgroep, 1815 JD Alkmaar, The Netherlands; 3 Department of Surgery, Medical Center Leeuwarden, 8934 AD Leeuwarden, The Netherlands; 4 Department of Epidemiology and Data Science, Amsterdam University Medical Centers, Vrije Universiteit Amsterdam, 1081 HV Amsterdam, The Netherlands; 5 Department of Medical Oncology, Amsterdam University Medical Centers, Cancer Center Amsterdam, 1081 HV Amsterdam, The Netherlands; 6 Department of Surgery, Amsterdam University Medical Centers, Cancer Center Amsterdam, 1081 HV Amsterdam, The Netherlands

**Keywords:** colorectal cancer (CRC), colorectal liver metastases (CRLMs), extra hepatic colorectal metastases, local treatment, thermal ablation, surgical resection, partial hepatectomy, stereotactic ablative radiotherapy, irreversible electroporation

## Abstract

**Simple Summary:**

For patients diagnosed with metastatic colorectal cancer, having colorectal liver metastases along with metastases in other organs may be deemed a contraindication for local treatment with curative intent. This observational study investigates whether administering local treatment to all metastatic sites could enhance overall survival rates. A total of 941 patients were included, among whom were 60 patients with metastases in both the liver and other organ(s). Our findings reveal that although patients with both liver and extrahepatic metastases exhibited lower survival rates compared to those with solely liver metastases, a survival plateau emerged after approximately 6.2 years. This implies that comprehensive local treatment of all metastatic sites might confer benefits for long-term survival. These insights could impact decision making regarding the scope of local treatment for patients with colorectal cancer that have metastases in multiple organs.

**Abstract:**

Background: The simultaneous presence of colorectal liver metastases (CRLMs) and extrahepatic metastases in patients with colorectal cancer (CRC) can be considered a relative contraindication for local treatment with curative intent. This study aims to assess the survival outcomes of patients with CRLMs and extrahepatic metastases after comprehensive local treatment of all metastatic sites. Methods: Patients with CRLMs who received local treatment of all metastatic sites were extracted from the prospective AmCORE registry database and subdivided into two groups: CRLM only vs. CRLM and extrahepatic metastasis. To address potential confounders, multivariate analysis was performed. The primary endpoint was overall survival (OS). Results: In total, 881 patients with CRLM only and 60 with CRLM and extrahepatic disease were included, and the median OS was 55.7 months vs. 42.7 months, respectively. Though OS was significantly lower in patients with concomitant extrahepatic metastases (HR 1.477; 95% CI 1.029–2.121; *p* = 0.033), the survival curve plateaued after 6.2 years. Extrahepatic manifestations were pulmonary (43.3%), peritoneal (16.7%) and non-regional lymph node metastases (10.0%). In patients with pulmonary and non-regional lymph node metastases, OS did not significantly differ from patients with CRLM-only disease; concomitant peritoneal metastases showed an inferior OS (HR 1.976; 95% CI 1.017–3.841, *p* = 0.041). Conclusions: In this comparative series, OS was inferior for patients with multi-organ metastatic CRC versus patients with CRLMs alone. Nonetheless, the long-term survival curve plateau seemed to justify local treatment in a subset of patients with multi-organ metastatic CRC, especially for patients with CRLMs and pulmonary or lymph node metastases.

## 1. Introduction

Colorectal cancer (CRC) represents 10% of the annual cancer incidence worldwide [1,2]. In 2020, it was the second leading cause of cancer-related deaths, with a mortality rate of 9.4%, affecting both males and females. The highest incidence of CRC-related mortality occurs in patients with metastatic disease [1,2]. During the course of their disease, 50–60% of patients develop distant metastases, with 80–90% of these cases concerning colorectal liver metastases (CRLMs) [3,4]. In cases where metastatic CRC is deemed unsuitable for local treatment, palliative systemic treatment alone has been shown to modestly improve 5-year overall survival (OS) from 3% to 11% [5,6,7,8,9]. If the CRLMs are primarily not eligible for local treatment, successful downstaging with induction chemotherapy can be achieved in up to 12.5% of patients. The 5-year OS increases up to 58% after local treatment of CRLMs in patients with liver only disease [5,10,11,12,13,14,15].

While liver metastases are often the primary manifestation of metastatic CRC, a difference in distribution of extrahepatic disease has been described for colon cancer (CC) and rectal cancer (RC). The distribution of extrahepatic disease in case of CC exists in most patients of peritoneal metastases (34%), pulmonary metastases (23%), and non-regional lymph node metastases (21%), whereas pulmonary metastases (37%) are predominant in RC, followed by non-regional lymph node metastases (21%) and peritoneal metastases (12%) [2,16].

Historically, the simultaneous presence of hepatic and extrahepatic metastases is considered to be a relative, if not an absolute, contraindication for local treatment with curative intent. This is mainly because chances of achieving cure were considered negligible. In 1995, Hellman et al. described the combination of localized cancer and distant metastases as an intermediate state of ‘oligo-metastatic-disease’, and curative local treatment should be considered if all visible cancer can be eradicated [17]. More recent research confirmed this hypothesis with presumed superior long-term survival in patients with CRLM and limited extrahepatic disease after comprehensive local treatment of all metastatic sites. Systemic treatment was added with the purpose of downstaging or it was used as induction therapy [4,14,18,19,20,21,22]. Considering the potential to achieve long-term disease control through local treatment of both CRLM and extrahepatic disease sites, the question arises: to what extent should patients with CRLM and extrahepatic disease be subjected to local treatment?

To our knowledge, no current guidelines state the extent and specific extrahepatic sites that should be considered for radical intent local treatment in patients with CRLM. The aim of this Amsterdam Colorectal Liver Met Registry (AmCORE)-based study is to evaluate survival outcomes of patients with simultaneously detected hepatic and extrahepatic disease.

## 2. Materials and Methods

This single-center study was performed at the Amsterdam University Medical Centers, The Netherlands, a tertiary referral center for hepatobiliary and gastrointestinal malignancies. The AmCORE database was accessed, containing prospectively maintained data of patients diagnosed with CRLM. The affiliated Institutional Review Board approved this study (METc 2021.0121). The STROBE guidelines were used to report and analyze the data for this observational study [23].

### 2.1. Data Collection and Patient Selection

Data were extracted from the prospectively maintained AmCORE database. Additional collection of data was performed retrospectively by searching the electronic patient files. Obtained data included patient, disease, and treatment characteristics, and follow-up information regarding OS and distant progression free survival (DPFS). According to the SIO- and DATECAN-initiated consensus guidelines, distant recurrence includes both new CRLMs at different sites and new extrahepatic metastases [24].

All included patients were diagnosed with CRLM, either with or without the presence of extrahepatic disease. After the diagnostic work-up, local treatment options for CRLMs were evaluated by a multidisciplinary team (MDT) attended by an (interventional) radiologist, oncological or hepatobiliary surgeon, medical oncologist, radiation oncologist, nuclear medicine physician, gastroenterologist, and pathologist. The MDT also determined the appropriate treatment approach regarding the extrahepatic disease. Patients who were not eligible for local treatment of CRLMs were excluded. In this study, there were no requirements regarding the local treatment approach for CRLM and extrahepatic disease sites. The treatment characteristics regarding the extrahepatic disease were not included in this study, as it was expected to vary significantly depending on the affected organs and the extent of the disease.

### 2.2. Diagnostic Work-up and Follow-up

Adhering to national and international guidelines, all patients diagnosed with CRC underwent diagnostic tests, including cross-sectional imaging, to detect possible metastatic disease [25,26,27]. Contrast-enhanced computed tomography (ceCT) of the chest and abdomen was available for every patient. If considered necessary by the local MDT, an additional 18 F-fluoro-2-deoxy-D-glucose positron emission tomography (18-FDG PET) scan was performed to detect possible distant metastases and local lymph node metastases. In case of CRLM, additional contrast-enhanced magnetic resonance imaging (ceMRI) with diffusion-weighted images (DWI) was performed to evaluate local treatment options [28,29].

During the first year after local treatment of CRLM, all patients received diagnostic ceCT of the chest and abdomen every 3 or 4 months, in accordance with national and international guidelines [25,26,27]. To detect residual disease after local treatment of CRLM and disease progression at any location, 18F-FDG PET-CT and/or liver ceMRI with DWI were used. Progressive disease was defined as a new solid mass or new 18F-FDG PET-CT avidity at any location. If uncertain, histopathological confirmation was obtained. During the second and third year after local treatment, ceCT of the chest and abdomen was performed every 6 months. During the fourth and fifth year after local treatment, a ceCT of the chest and abdomen was performed every year.

### 2.3. Statistical Analysis

Baseline characteristics were compared between two groups: patients with CRLM-only disease and patients with CRLM and extrahepatic disease. Categorical variables are reported as frequencies (in percentages; %). The Fisher’s exact test for dichotomous variables and Pearson’s chi-squared test for categorical variables were used to compare characteristics in both groups. Continuous variables are reported as mean with standard deviation (SD), if normally distributed, and as median with interquartile range (IQR), if non-normally distributed. To compare both groups, the independent samples *t*-test was used when variables were normally distributed and the Mann–Whitney U test was used when they were when non-normally distributed.

Kaplan–Meier curves with the log-rank test were used to estimate and compare OS and DPFS. The primary endpoint is defined as time-to-event (death) from the date of diagnosis of first CRLM. Data were right-censored for those still alive during last follow-up. OS was reviewed using Cox proportional hazards regression models, accounting for potential confounders in multivariable analysis. Variables with *p* < 0.050 in univariable analysis were included in the multivariable analysis. Variables were removed step by step, using backward selection to identify significant confounders (*p* < 0.050). Hazard ratios (HRs) and 95% confidence intervals (95% CIs) were reported. When a change of >10% was found for the regression coefficient in the corrected model, variables were considered actual confounders.

A biostatistician supported the statistical analyses (B.I. Lissenberg-Witte). The statistical analyses were conducted using SPSS Version 28.0 (IBM Corp, Armonk, NY, USA) [30] and R Version 4.2.1 (R Foundation, Vienna, Austria) [31].

## 3. Results

**Figure 1:**
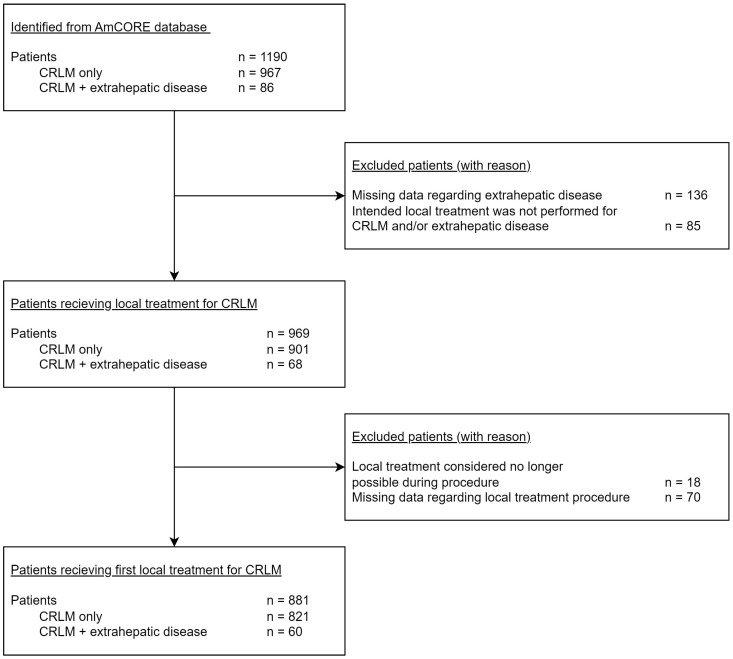
Flowchart of included and excluded patients.

Between January 2000 and July 2023, a total of 1190 patients with CRLM were identified from the AmCORE database. Eighty-six of these patients were also diagnosed with extrahepatic disease at time of the first CRLM diagnosis. See the flowchart for a more detailed description of patient selection (Figure 1).

### 3.1. Patient and Disease Characteristics

Baseline patient-, disease-, and treatment-related characteristics are presented in Table 1. A total of 881 patients were included: 821 patients with CRLM-only disease and 60 patients with extrahepatic disease at time of diagnosis of the CRLM. Sixty-six percent of patients were male, with no statistical difference in the distribution of gender between both groups. Mean age was 65.6 years (SD 11.3). The median follow-up duration of this patient population was 29.4 months.

The primary tumor location was distributed as follows: right-sided in 24.1%, left-sided in 42.0%, and in the rectum in 33.8%. Patients with CRLM and extrahepatic disease showed a significantly higher number of RAS mutations compared to RAS wildtype (*p* = 0.050). Mutation status including RAS and BRAF mutations and MMR status was frequently unknown (73.9%, 83.1%, and 84.9%, respectively), as shown in Table 1. For patients with known RAS mutational status, RAS mutations were found in 66.8% of patients with CRLMs and extrahepatic metastases, compared to 40.5% in patients without extrahepatic disease.

Patients with CRLM were treated with either resection (34.6%); thermal ablation (30.1%) (including microwave ablation (MWA) and radiofrequency ablation (RFA)); a combination of resection and thermal ablation (26.7%); irreversible electroporation (IRE) (3.6%); or stereotactic ablative body radiotherapy (SABR) (5.0%). The local treatment of extrahepatic metastatic sites was performed as follows: SABR for pulmonary metastases, surgical resection for lymph node metastases, and a combination of hyperthermic intraperitoneal chemotherapy (HIPEC) and cytoreductive surgery (CRS) for peritoneal metastases. For patients with pulmonary metastases, no major complications were reported in relation to the SABR treatment. After surgical resection of lymph node metastases, no major complications were reported related to the resection of the lymph node(s). In two patients, major complications did occur related to a HIPEC-CRS treatment. One patient experienced a postoperative paralytic ileus, leading to an extended hospital stay. Another patient developed pulmonary embolisms and a duodenal ulcer necessitating coiling of the gastroduodenal artery.

**Figure 2:**
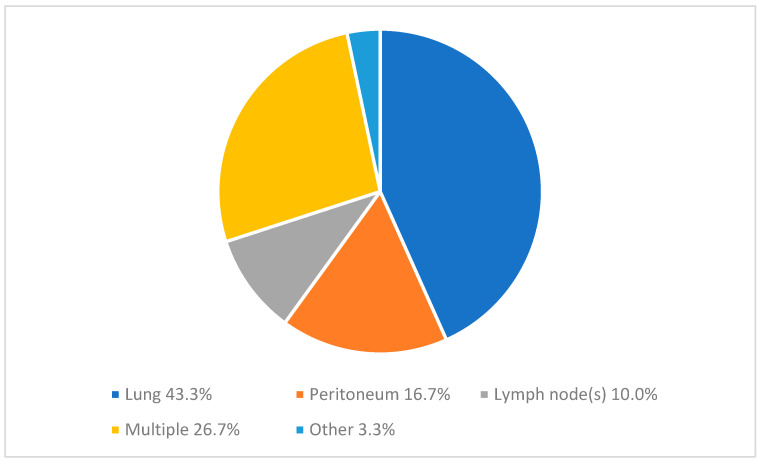
Organ(s) affected by extrahepatic disease. Reported as number of patients (%). Multiple = more than one organ is affected by metastatic disease; other = spleen and adrenal gland. Lymph node metastases were located in the lung hilum (1×), liver hilum (1×), spleen (2×), supra clavicular (1×), para-cardiac (1×), para-aortic (3×), para-iliac (2×), retro peritoneal (1×), and unknown (2×) regions due to no clear description in the available radiology report.

Figure 2 demonstrates an overview of the location of extrahepatic disease. Distribution of affected organ(s) is as follows: 43.3% lung, 16.7% peritoneum, 10% non-regional lymph node(s), 26.7% multiple organs, and 3.3% other (spleen and adrenal gland). A comparison of the specific location of extrahepatic disease per primary tumor location did not show any significant differences (Table 2).

### 3.2. Overall Survival and Disease-Free Survival

**Figure 3:**
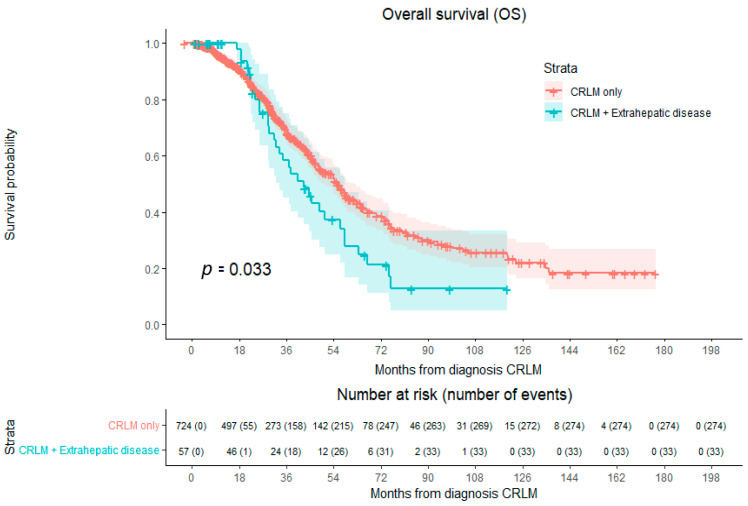
Kaplan–Meier curves of overall survival (OS), *p* = 0.033. Compared with log-rank test. Red indicates patients with CRLM-only disease. Blue indicates patients with extrahepatic disease at time of first diagnosis CRLM.

Patients with CRLMs and extrahepatic metastases showed a significantly lower OS compared to patients with CRLM-only disease (HR 1.477; 95% CI 1.029–2.121; *p* = 0.033) (Figure 3). Median OS of the entire cohort was 54.2 months (95% CI 49.372–59.047). Median OS in patients with CRLM-only disease was 55.7 months (95% CI 50.277–61.099), with a 1-year OS of 94.9%, 3-year OS of 69.4%, and 5-year OS of 44.7%. The overall survival curve shows a plateau survival rate of approximately 10% beyond 6 years. In patients with CRLM and extrahepatic disease, the median OS was 42.7 months (95% CI 32.439–52.982), with a 1-year OS of 100.0%, 3-year OS of 58.3%, and 5-year OS of 28.0%.

**Figure 4:**
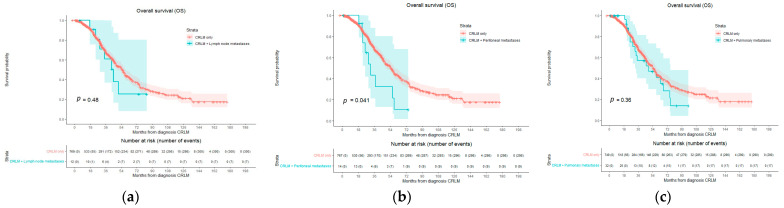
Kaplan–Meier curves of overall survival (OS). Compared with log-rank test. OS per patient comparing CRLM only to (**a**) CRLM with pulmonary metastasis (*p* = 0.363), to (**b**) CRLM with peritoneal metastasis (*p* = 0.041), and to (**c**) comparing CRLM only and non-regional lymph node metastasis (*p* = 0.483). Number of events are per patient.

Subgroup analyses of OS in patients with CRLMs and pulmonary metastases showed a median OS of 48.5 months, with a 1-year OS of 100.0%, 3-year OS of 57.1%, and 5-year OS of 10.9%. Figure 4a shows no significant difference in OS compared to patients with CRLM-only disease (HR 1.255; 95% CI 0.769–2.049; *p* = 0.363). In patients with CRLMs and peritoneal metastases, the median OS was 31.5 months (Figure 4b), with a 1-, 3-, and 5-year OS of 100.0%, 32.3%, and 10.8%, respectively. This is significantly lower compared to OS in patients with CRLM-only disease (HR 1.976; 95% CI 1.017–3.841, *p* = 0.041). OS curves for patients with CRLM-only disease and CRLM with non-regional lymph node metastases are presented in Figure 4c. There was no statistical difference found regarding OS (*p* = 0.483) with a HR of 1.308 (95% CI 0.618–2.769). Median OS was 44.7 months, with a 1-, 3-, and 5-year OS of 100.0%, 60.6%, and 25.3%, respectively.

**Figure 5:**
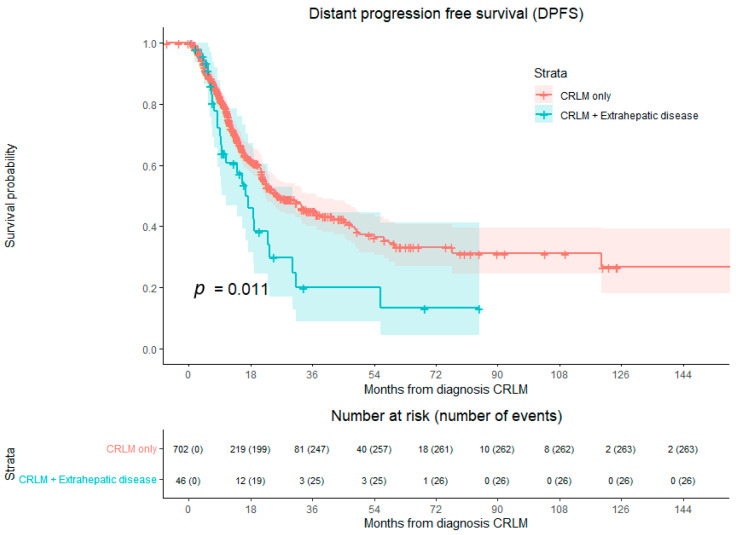
Kaplan–Meier curves of distant progression-free survival (DPFS), *p* = 0.011. Compared with log-rank test. Red indicates patients with CRLM-only disease. Blue indicates patients with extrahepatic disease at time of first diagnosis of CRLM. Successfully retreated local recurrences were not considered as an event in this analysis.

During follow-up, the median DPFS for patients with CRLM-only disease was 25.955 months (Figure 5). In patients with CRLMs and extrahepatic metastases, the median DPFS was 16.559 months. Compared to CRLMs with extrahepatic metastases, DPFS was superior for CRLM-only disease (*p* = 0.011) with an HR for CRLM of only 1.677 (CI 95% 1.120–2.510).

Potentially associated variables are presented in Table 3. Association of ASA (American Society of Anesthesiologist) physical status (*p* ≤ 0.001), primary tumor location (*p* = 0.068), synchronicity of first-diagnosis CRLM (*p* ≤ 0.001), and type of local treatment (*p* = 0.056) with OS was identified in univariable analysis. Hereafter, the potential confounding influence of these variables was analyzed with multivariable analysis. After adjusting for the confounders of ASA physical status (*p* ≤ 0.001) and synchronicity of first-diagnosis CRLM (*p* ≤ 0.001), the HR for OS in patients with CRLM-only disease compared to patients with CRLM and extrahepatic metastases was 1.512 (95% CI 1.011–2.260, *p* = 0.044). Therefore, these variables were not considered actual confounders.

## 4. Discussion

This study showed an inferior OS when extrahepatic metastases were present at time of first diagnosis of CRLMs (HR 1.512). A 5-year OS of 44.7% was found in the group with CRLM-only disease compared to a 5-year OS of 28.0% for patients diagnosed with CRLM and extrahepatic disease. The survival curve shows a plateau survival rate of approximately 10–15% beyond 6 years, suggestive of long-term disease control in a subset of patients with multi-organ metastatic CRC. Pulmonary metastases (43.3%), peritoneal metastases (16.7%), and non-regional lymph node metastases (10%) were the three most common locations for extrahepatic disease in this cohort. The 5-year OS for patients diagnosed with pulmonary metastases and non-regional lymph node metastases was not significantly different from the group of patients with CRLM-only disease. The group of patients diagnosed with peritoneal metastases showed a significantly worse 5-year OS compared to patients with CRLM-only disease. The comparable OS after local treatment of CRLM alone versus after CRLM and pulmonary or lymph node metastases, as well as the worse OS in patients with peritoneal metastases, underscores the importance of considering the location of extrahepatic metastases as a prognostic and potentially predictive parameter to select patients that might benefit from it.

The current literature discussing which strategy is preferable in patients with multi-organ metastatic CRC is limited, and the consensus to what extent local treatment should be performed is lacking. No randomized controlled studies previously compared systemic therapy alone to radical intent local treatment in patients with hepatic and extrahepatic CRC metastases, and no robust data exist to recommend local treatment for specific extrahepatic metastatic sites. No guidelines state which treatment techniques should be used and to what extent multi-organ metastatic disease should be considered amenable for radical intent local treatment [25,26,27].

In this study, the difference in OS between patients who received local treatment for CRLM only and CRLM in combination with all sites of extrahepatic disease was comparable to the results from previous retrospective studies, with a 5-year OS varying from 19 to 26% [33,34]. Byam et al. previously showed a superior median OS for surgical resection of all metastatic sites plus systemic chemotherapy over chemotherapy alone (24 months vs. 13 months, *p* = 0.01) [35]. In addition, the current literature describes that after complete resection of extrahepatic metastatic sites, 5-year OS rates of 40–68% can be achieved, comparable to CRLM-only disease [19,33,34,36]. The results of the ongoing multinational randomized controlled ORCHESTRA trial (registered as NCT01792934) will hopefully provide clarity on the added value of maximal tumor debulking combined with systemic treatment versus systemic treatment alone in patients with multi-organ metastatic CRC [37].

The distribution of extrahepatic disease found in this study corresponds to the findings in previously reported results [33,35]. When comparing the specific location of extrahepatic disease per primary tumor location, no statistical difference was found. Based on the previous literature, a different distribution pattern could have been expected due to the differences in venous drainage based on the anatomical location of CC and RC [2,27].

In this study cohort, all pulmonary metastases were treated with SABR, in accordance with the European guidelines [25,27]. After combined resection of pulmonary and hepatic metastases in carefully selected patients, the current literature reports a 5-year OS of 27–60% [19,38,39,40,41,42,43,44]. Previous studies have been published to evaluate different local treatment techniques for pulmonary metastases; they show that an increasing subset of patients were offered surgical resection, SABR, or thermal ablation with similar results for all three treatment techniques regarding treatment outcomes and technical efficacy [24,35,45,46,47,48,49,50,51]. Nevertheless, a systematic review by Schlijper et al. states that due to the lack of phase III trials, no firm conclusions can be drawn regarding the optimal treatment technique to curatively treat colorectal pulmonary metastases [22].

Regarding the local treatment of lymph node metastases, one systematic review showed a 5-year OS of 17% in patients undergoing resection of only portacaval lymph node metastases combined with surgical resection of CRLMs [33]. A more recent retrospective study described a significantly increased OS for patients with oligo non-regional lymph node metastases after local treatment (surgical resection or SABR), compared to palliative treatment alone (73.49 months vs. 23.22 months, *p* = 0.01) [52]. Resection or ablative therapy (thermal ablation and SABR) for non-regional lymph node metastases have shown long-term survival benefits in selected cases [27,53]. When comparing the results from this study after local treatment of CRLMs and non-regional lymph node metastases to the current literature, several differences were found regarding OS. This could be explained by the heterogeneity of the location of lymph node metastases in this cohort, especially when considering that the recent literature found a different impact on OS for varied locations of non-regional lymph node metastases [52,53,54].

The outcomes of patients with CRLMs and peritoneal metastases evaluated in this study correlate to the results of previous studies, where 5-year OS varied from 8 to 25% [33,55]. HIPEC combined with CRS is considered a standard of care to treat limited peritoneal metastases. Here, a peritoneal cancer index of < 20 is considered to be limited and therefore signifies a potential curative disease [26,27]. A systematic review by Polderdijk et al. states that a 5-year OS rate of 25% can be achieved when combining local treatment of limited CRLM and CRS-HIPEC in selected cases with a previous response to systemic therapy [55]. Current national guidelines advise a maximum of three CRLMs present when considering a combined treatment strategy with local treatment of CRLM and CRS-HIPEC of peritoneal metastases [26]. However, studies outlining clear recommendations regarding the cutoff values for the number of CRLMs that may be present to consider treatment for extra-peritoneal metastases is lacking.

The relatively high number of patients included in the study allowed for a statistical analysis with adequate power. However, because the number of patients with CRLM and extrahepatic disease was relatively small, conclusions pertaining to the effects of metastatic location on OS cannot extend beyond a discernible trend. Additionally, this study’s non-randomized design is a significant limitation as it introduces selection and immortality time bias. Even though a multivariable analysis was conducted to account for potential confounders, complete elimination of residual confounders is not guaranteed. The inclusion of patients treated over a time span exceeding 20 years may have introduced some population or historical bias because the advancements in both systemic and local treatment techniques were not considered [15].

The RAS and BRAF mutation status was frequently unknown in this study, as it was not routinely performed as a standard of care for patients with metastatic CRC in the past and is still not routinely performed for patients who do not receive systemic therapy. Additionally, MMR status was not routinely determined in the past, but has been implemented during the time of patients’ inclusion in this cohort study. As these variables were not included in the uni- and multivariate analyses, the potential confounding effect on OS remains unknown. The significantly higher number of RAS mutations found in the group with CRLM, as well as extrahepatic metastases compared to patients with CRLM only, represents a residual confounder as RAS mutations are known to be associated with a worse prognosis.

This study did not clarify the possible confounding effects on OS regarding the administration of systemic treatment in combination with local treatment for all metastatic sites. It is likely that patients with stable or progressive disease during systemic treatment did not receive local treatment, resulting in a worse prognosis. This might also be true in relation to patients that were not considered eligible for systemic treatment upfront due to their overall condition, or regarding patients with right-sided CRC generally having a worse prognosis compared to patients with left-sided CRC and being excluded from anti-EGFR treatment [27]. Although the added value of neoadjuvant or periprocedural chemotherapy remains unclear, the fact that not all patients in the CRLM-only group received chemotherapy prior to local treatment represents another potential confounder.

## 5. Conclusions

In conclusion, in this comparative series, OS was inferior in patients with multi-organ metastatic CRC versus patients with CRLM alone. Nonetheless, the long-term survival curve plateau seems to justify local treatment in a subset of patients with multi-organ metastatic CRC, especially for patients with CRLMs and pulmonary or lymph node metastases.

## Figures and Tables

**Table 1:** Patient-, disease-, and treatment-related characteristics.

		Total n = 881	CRLM Only n = 821	CRLM and Extrahepatic n = 60	*p*-Value
**Patient-related characteristics**					
Gender	Male	66.0	66.9	54.2	
	Female	34.0	33.1	45.8	0.063 ^a^
Age (years)	Mean (SD)	65.6 (11.3)	65.8 (11.3)	64.0 (10.7)	0.261 ^b^
ASA physical status	1	6.0	6.3	2.0	
	2	70.3	70.3	70.6	
	3	23.3	23.0	27.5	
	4	0.4	0.4	0.0	0.549 ^c^
Comorbidities	None	48.1	48.1	48.1	
	Minimal	38.6	38.1	44.2	
	Major	13.3	13.7	7.7	0.407 ^c^
BMI (kg/cm^2^)	Mean (SD)	26.1 (4.4)	26.1 (4.4)	26.0 (4.6)	0.964 ^b^
**Disease-related characteristics**					
Primary tumor location	Rectum	33.8	24.3	34.5	
	Right-sided colon	24.1	33.8	22.4	
	Left-sided colon	42.0	41.9	43.1	0.950 ^c^
Molecular profile	RASwt/mut/unknown	9.6/7.3/83.1	9.7/6.6/83.7	8.3/16.7/75.0	0.050 ^c^
	BRAFwt/mut/unknown	14.0/1.1/84.9	13.8/1.0/85.3	16.7/3.3/80.0	0.208 ^c^
	MSS/MSI/unknown	25.7/0.5/73.9	25.6/0.6/73.9	26.7/0.0/73.3	0.581 ^c^
Diagnosis of CRLM	Synchronous ^d^	46.9	47.2	42.9	
	Early metachronous ^e^	22.6	23.0	17.9	
	Late metachronous ^f^	30.5	29.8	39.3	0.306 ^c^
**Treatment-related characteristics**					
Type of local treatment	Resection	34.6	33.6	48.3	
	TA	30.1	30.6	23.3	
	Resection and TA	26.7	27.3	18.3	
	IRE	3.6	3.7	3.3	
	SABR	5.0	4.9	6.7	0.163 ^c^

Categorical variables are reported as number of patients (%); continuous variables are reported as mean (SD). ^a^ = Fisher’s exact test; ^b^ = independent *t*-test; ^c^ = Pearson’s chi-squared test; RAS = rat sarcoma viral oncogene homolog; BRAF = V-raf murine sarcoma viral oncogene homolog B; wt = wildtype; mut = mutation; MSS = microsatellite stability; MSI = microsatellite instability; ^d^ = synchronous—within 8 weeks of diagnosis primary tumor; ^e^ = early metachronous—within 1 year after diagnosis primary tumor; ^f^ = late metachronous, ≥1 year after diagnosis primary tumor [32]; ASA = American Society of Anesthesiologists score; TA= thermal ablation—includes microwave ablation (MWA) and radiofrequency ablation (RFA); IRE = irreversible electroporation; SABR = stereotactic body radiation.

**Table 2:** Location of extrahepatic disease per primary tumor location.

		RC n = 20	Left-Sided CR n = 25	Right-Sided CR n = 13	*p*-Value
Extrahepatic disease	Lung	12 (60)	8 (32)	5 (39)	
	Non-regional lymph node(s)	1 (5)	2 (8)	2 (15)	
	Peritoneum	0	7 (28)	2 (15)	
	Multiple	4 (20)	2 (8)	3 (23)	
	Other	3 (15)	6 (24)	1 (8)	0.161 ^a^

Reported as number of patients (%); ^a^ = Pearson’s chi-squared test. Total number of patients is 58, because the information regarding the primary tumor location is missing in the data of two patients.

**Table 3:** Uni- and multivariable Cox regression analyses to detect association of variables to overall survival (OS).

		Univariable Analysis		Multivariable Analysis	
		HR (95% CI)	*p*-Value	HR (95% CI)	*p*-Value
Extrahepatic disease at first diagnosis of CRLM	No Yes	Reference 1.477 (1.029–2.121)	0.035	Reference 1.512 (1.011–2.260)	0.044
**Patient-related characteristics**					
Gender	Male	Reference	0.221		
	Female	0.861 (0.677–1.095)			
Age					
ASA physical status	1	Reference	<0.001	Reference	<0.001
	2	0.920 (0.559–1.513)		1.058 (0.601–1.864)	
	3	1.498 (0.884–2.539)		1.649 (0.907–3.000)	
	4	9.958 (1.278–77.610)		18.836 (2.372–149.565)	
Comorbidities	None	Reference	0.821		
	Minimal	1.015 (0.707–1.457)			
	Major	0.801 (0.396–1.621)			
BMI		1.001 (0.974–1.029)	0.916		
**Disease-related characteristics**					
Primary tumor location	Rectum	Reference	0.021	Reference	0.068
	Right-sided colon	0.793 (0.612–1.029)		0.862 (0.642–1.157)	
	Left-sided colon	1.187 (0.883–1.595)		1.276 (0.909–1.791)	
Diagnosis of CRLM	Synchronous ^a^	Reference	<0.001	Reference	<0.001
	Early metachronous ^b^	1.711 (1.294–2.264)		1.752 (1.307–2.351)	
	Late metachronous ^c^	1.015 (0.765–1.349)		0.908 (0.666–1.239)	
**Treatment-related characteristics**					
Type of local treatment	Resection	Reference	<0.001	Reference	0.056
	TA	1.166 (0.868–1.567)		1.272 (0.924–1.751)	
	Resection and TA	1.061 (0.789–1.427)		1.035 (0.748–1.432)	
	IRE	1.466 (0.803–2.675)		1.560 (0.847–2.873)	
	SABR	2.597 (1.758–3.837)		2.307 (1.215–4.383)	

Categorical variables are reported as number of patients (%); continuous variables are reported as mean (SD). RAS = rat sarcoma viral oncogene homolog; BRAF = V-raf murine sarcoma viral oncogene homolog B; wt = wildtype; mut = mutation; MSS = microsatellite stability; MSI = microsatellite instability; ^a^ = synchronous—within 8 weeks of diagnosis primary tumor; ^b^ = early metachronous—within 1 year after diagnosis primary tumor; ^c^ = late metachronous—≥1 year after diagnosis primary tumor [32]; ASA = American Society of Anesthesiologists score; TA = thermal ablation—includes microwave ablation (MWA) and radiofrequency ablation (RFA); IRE = irreversible electroporation; SABR = stereotactic body radiation.

## Data Availability

The data presented in this study are available upon request from the corresponding author.
